# Hormonal Therapy and Risk of Breast Cancer in Mexican Women

**DOI:** 10.1371/journal.pone.0079695

**Published:** 2013-11-15

**Authors:** Amina Amadou, Alban Fabre, Gabriela Torres-Mejía, Carolina Ortega-Olvera, Angélica Angeles-Llerenas, Fiona McKenzie, Carine Biessy, Pierre Hainaut, Isabelle Romieu

**Affiliations:** 1 Nutrition and Metabolism Section/Nutritional Epidemiology Group, International Agency for Research on Cancer, Lyon, France; 2 National Institute of Public Health, Cuernavaca, Morelos, Mexico; 3 International Prevention Research Institute, Lyon, France; 4 Nutrition, Hormones and Women’s Health Institute Gustave Roussy, INSERM U1018, Paris, France; Nanjing Medical University, China

## Abstract

The use of hormonal therapies, including hormonal contraceptives (HC) and postmenopausal hormone replacement therapy (HRT) have been shown to influence breast cancer (BC) risk. However, the variations of these effects among populations and ethnic groups are not completely documented, especially among Hispanic women. We evaluated the association between HC and premenopausal BC risk, and between HRT and postmenopausal BC risk in Mexican women. Data from a Mexican multi-center population-based case–control study ofwomen aged 35 to 69 years were analysed. A total of 1000 cases and 1074 matched controls were recruited between 2004 and 2007. Information on hormonal therapy was collected through a structured questionnaire. Results were analysed using conditional logistic regression models. Overall, HC were used by 422/891 (47.3%) premenopausal women and HRT was used by 220/1117 (19.7%) postmenopausal women. For HC, odds ratios (ORs) for BC were 1.11 (95% confidence interval (CI): 0.82, 1.49) for current users and 1.68 (95% CI: 0.67, 4.21) for ever-users. No clear effect of duration of use was observed. For HRT, the OR for BC was significantly increased in ever users (OR: 1.45; 95% CI: 1.01, 2.08). A non-significant increased risk was observed for combined estrogen/progestin, (OR =  1.85; 95% CI: 0.84, 4.07) whereas no effect was observed for the use of estrogen alone (OR = 1.14; 95% CI: 0.68, 1.91). Our results indicate that, HC had a non-significant effect on the risk of pre-menopausal BC, but suggested that injected contraceptives may slightly increase the risk, whereas HRT had a significant effect on post-menopausal BC in this population. This study provides new information about the effects of HC and HRT on BC risk in a Mexican population, which may be of relevance for the population of Latin America as a whole.

## Introduction

Risk factors for breast cancer (BC) show variable associations with the disease according to ethnicity/race. However, these associations are still incompletely documented in many populations, including Hispanic populations [1]–[3]. In the United States (US), the lower incidence of BC in Hispanics compared with Caucasians is partially explained by difference in the distribution of BC risk factors such as late age at menarche, early age at first full-term pregnancy and large number of children [4], [5]. Together, these reproductive factors account for less than 20% of the difference between the two ethnic groups for postmenopausal women [6]. In contrast, Hispanic women in the US have higher prevalence of obesity and physical inactivity than Caucasian, two factors associated with increased risk of BC [4].

The use of hormonal therapies including hormonal contraceptives (HC) and post-menopausal hormone replacement therapy (HRT) has been shown to be associated with increased risk of BC. HC are among the most commonly used drugs worldwide. Several large epidemiological studies that have assessed the effect of HC on the risk of BC have reported an increased risk of premenopausal BC [7]–[11]. For HRT, evidence from randomized controlled trials and observational studies has shown that women using HRT are at an increased risk of BC [9], [12]–[18]. Moreover, the risk of BC associated with HRT is larger for users of combined HRT than for users of estrogen-only therapy [9], [19]–[22]. These results, as well as data on the risk of several other cancers, have led the International Agency for Research on Cancer (IARC) to classify combined estrogen-progestogen contraceptives and combined HRT as carcinogenic for humans [23], [24]. However, the majority of these studies were based on Caucasian women. Although the evidence that hormonal therapies influence BC development among Caucasian women is extensive, less is known about these relationships among Hispanic women [20], [25]. There are no studies of Mexican women living in México, and given the lack of public awareness [26], the use of hormonal therapies will probably continue to increase over the next years. Therefore, there is an urgent need to know how hormonal therapies affect BC in a Hispanic population living in Latin America.

In this study, we have used data from a multi-center population-based case-control study conducted in Mexico to assess the effects of hormonal therapy on the risk of BC in a non-US Hispanic population. We have investigated the association between the use of HC and premenopausal BC risk, and between the use of HRT and the risk of postmenopausal BC in Mexican women. In addition, we have conducted specific analyses to investigate the effects of duration of use and types of hormonal therapies on the risk of BC.

## Materials and Methods

### Ethics statement

Cases and controls provided written informed consent to participate in the study. The study protocol and data collection instruments were reviewed and approved by the Institutional Review Board at the National Institute of Public Health.

### Study population

A multi-center population-based case-control study was designed to examine predictors of risk for BC among Mexican women. In this study 1000 cases and 1074 controls, pre or postmenopausal women aged 35 to 69 years, were recruited between January 2004 and December 2007 from three regions in Mexico and their surrounding metropolitan areas (Mexico City, Monterrey, and Veracruz). Participants were resident from one of these regions during at least 5 years prior to recruitment in the study.

Cases were identified by trained field staff at 12 hospitals from major health care institutions in Mexico: the Mexican Institute of Social Security (Instituto Mexicano del Seguro Social, IMSS, six hospitals), the Social Security and Services Institute for State Employees (Instituto de Seguridad y Servicios Sociales de los Trabajadores del Estado, ISSSTE, two hospitals), and the Ministry of Health (Secretarıade Salud, SS, four hospitals). Inclusion criteria included: (a) women with a new histologically confirmed diagnosis of in situ or invasive primary BC, regardless of the stage of disease (median time to inclusion  =  3 days from the day of diagnosis); (b) women who were not previously treated with radiotherapy, chemotherapy, or anti-estrogen drugs such as tamoxifen during the previous 6 months; (c) women who were not currently using aromatase inhibitors and (d) women who were not pregnant. Two cases that were using anti-estrogens were not included because of the effect of the latter on breast density. One HIV (human immunodeficiency virus) positive woman was excluded. The response rate for cases was 95.5% for Mexico City, 94.4% for Monterrey and 97.4% for Veracruz. Controls were selected based on a probabilistic multistage design as described elsewhere [27] and were frequency-matched to the cases according to 5-year age groups, health care institution and region. The response rate for controls was 87.4% for Mexico City, 90.1% for Monterrey and 97.6% for Veracruz. An appointment was scheduled for each woman to attend the hospital for anthropometric measurements, mammography and a blood sampling. Detailed information regarding the estimation of the proportion of genetic ancestry (European, African, and Indigenous American) at an individual level was determined as described by Fejerman *et al*. [28]. Socioeconomic status (SES) was defined using an index for the Mexican population developed by Bronfman *et al*. [29]. This variable was categorized as tertiles to indicate low, medium, and high SES. Information on the type of menopause (natural or surgical) was obtained by questionnaire. Natural menopause was defined as 12 consecutive months of amenorrhea without an obvious cause.

### Classification of use of HC and HRT

Women were individually interviewed about HC and HRT using the following questions: (1) Have you ever taken HC or HRT? (2) If yes, how old were you when you started taking HC or HRT? (3) If yes, how old were you when you stopped taking such therapy (last used)? (4) If yes, how long in total did you take each therapy? (5) What type of treatment did you use? Women reported their age at starting and stopping HC and HRT in years and months. Time since last use was calculated as time since last reported use of HC in premenopausal women or of HRT in postmenopausal women and duration of use was calculated by adding together the total amount of time that HC or HRT use was reported. In premenopausal women, variables were then constructed as follows: ever use (ever versus never), time since last use (≥ 10 years, < 10 years, recent, never), total duration of use (≥ 10 years, < 10 years, never), and type of treatment (none, oral, injected or transdermal, more than one treatment). In postmenopausal women, categories were created for users of HRT as follows: ever use (ever versus never), time since last use (past, recent, never), total duration of use (≥ 5 years, < 5 years, never), and type of treatment (none, estrogen alone, estrogen/progestin). We defined recent use as the reported use of HC or HRT during the year prior to the date of interview. The categories used for these variables were defined a priori, taking into account the sample size in the relevant categories.

### Statistical analysis

We used Chi^2^ or Fisher’s exact tests to compare frequency distributions of characteristics of HC users and non-users in the premenopausal population and of HRT users and non-users in the postmenopausal population. Conditional logistic regression models were used to examine these associations. Multivariate adjusted odds ratios (OR) and corresponding 95% confidence intervals (CI) were estimated, adjusting for age at diagnosis (continuous), SES (high/medium/low), body mass index (BMI) (kg/m^2^, continuous), family history of BC (yes/no), parity (continuous), age at first full term pregnancy (continuous), age at menarche (continuous), total duration of breastfeeding (continuous), and diabetes (yes/no). In addition, we further adjusted for genetic admixture (genetic ancestry). We then stratified the categories (ever user or never user) by BMI and genetic ancestry (as defined based on evaluation of genetic admixture) [28] in order to evaluate potential interactions between hormonal therapy and BMI or genetic ancestry. BMI was stratified into two categories: normal weight (BMI < 25 kg/m^2^) and overweight (BMI ≥ 25 kg/m^2^). Indigenous American genetic ancestry was modeled as a categorical variable (0–50%, 51–75%, 76–100%). Log-likelihood tests were used to evaluate linear trends and interaction terms. All tests of statistical significance were two-sided and p ≤ 0.05 was considered as significant. All statistical analyses were performed using SAS software (version 9.0, SAS Institute, Inc., Cary, NC).

## Results

### Characteristics of the study population

The main characteristics of the study population are shown in Table 1. One thousand cases (415 premenopausal and 585 postmenopausal women) and 1074 controls (476 premenopausal and 598 postmenopausal women) were included in the study. Overall, information on the use of hormonal therapies was available for 2008 participants. A total of 422/891 (47.3%) premenopausal women used HC and 220/1117 (19.7%) postmenopausal women used HRT. In premenopausal women, compared with women who had never used HC, users were more likely to be younger at first pregnancy, to be parous, and to have a long duration of breastfeeding. In postmenopausal women, compared with women who never used HRT, users were more likely to have fewer children, to have a higher level of SES and to have a long duration of breastfeeding.

**Table 1:** Demographic and risk factor characteristics of the study population, according to the use of hormonal contraceptive and hormone replacement therapy.

	Premenopausal			Postmenopausal		
Characteristics	HC non-users	HC users	P-value	HRT non-users	HRT users	P-value
Age (years)						
< 40	314 (66.9)	244 (57.8)		32 (3.6)	5 (2.3)	
40–54	150 (32)	173 (41)	0.016	294 (32.8)	81 (36.8)	0.167
55–64	4 (0.9)	5 (1.2)		403 (44.9)	105 (47.7)	
≥ 65	1 (0.2)	-		168 (18.7)	29 (13.2)	
BMI (kg/m2)						
< 25	96 (20.5)	78 (18.5)		120 (13.4)	34 (15.5)	
25–29.9	197 (42.0)	185 (43.9)	0.732	335 (37.3)	92 (41.8)	0.217
≥ 30	176 (37.5)	159 (37.7)		442 (49.3)	94 (42.7)	
Age at menarche						
< 13	222 (47.3)	204 (48.3)	0.764	344 (38.4)	99 (45)	0.071
≥ 13	247 (52.7)	218 (51.7)		553 (61.6)	121 (55)	
Family history of breast cancer						
No	440 (93.8)	402 (95.3)	0.345	851 (94.9)	212 (96.4)	0.355
yes	29 (6.2)	20 (4.7)		46 (5.1)	8 (3.6)	
Socio economic status						
Low	139 (29.6)	130 (30.8)		341 (38)	45 (20.4)	
Medium	141 (30.1)	126 (29.8)	0.925	264 (29.4)	59 (26.8)	< 0.001
High	189 (40.3)	166 (39.3)		292 (32.6)	116 (52.7)	
Age at 1rst full term pregnancy						
Nulliparous	67 (14.3)	18 (4.3)		68 (7.6)	25 (11.3)	
< 22	188 (40.1)	242 (57.3)	< 0.001	455 (50.7)	105 (47.7)	0.215
≥ 22	209 (44.5)	156 (36.9)		365 (40.7)	86 (39.1)	
Missing	5 (1.1)	6 (1.4)		9 (1)	4 (1.8)	
Number of full term pregnancies						
0	67 (14.3)	18 (4.3)		68 (7.6)	25 (11.4)	
1−2	203 (43.3)	151 (35.8)	< 0.001	210 (23.4)	60 (27.3)	0.087
≥ 3	195 (41.6)	253 (59.9)		615 (68.6)	133 (60.4)	
Missing	4 (0.8)	-		4 (0.4)	2 (0.9)	
Breastfeeding						
Nulliparous	67 (14.3)	18 (4.3)		68 (7.6)	25 (11.3)	
< 12	43 (9.2)	49 (11.6)	< 0.001	92 (10.3)	31 (14.1)	0.010
≥ 12	124 (26.4)	142 (33.6)		168 (18.7)	51 (23.2)	
Missing	235 (50.1)	213 (50.5)		569 (63.4)	113 (51.4)	
Native ancestry						
0–50%	113 (24.1)	117 (27.7)		244 (27.2)	64 (29.1)	
51–75%	205 (43.7)	199 (47.2)	0.093	367 (40.9)	90 (40.9)	0.047
76–100%	106 (22.6)	80 (18.9)		199 (22.2)	34 (15.5)	
Missing	45 (9.6)	26 (6.2)		87 (9.7)	32 (14.5)	

P-value (Chi^2^ or Fisher’s exact tests) for the difference between HC non-users and users in premenopausal, and HRT non-users and users in postmenopausal women.

### HC use and breast cancer risk in pre-menopausal women

The association between use of HC and BC risk was non-significant (OR  =  1.11; 95% CI: 0.82, 1.49 for ever versus never users) (Table 2). Recent users had a non-significant increase in BC risk (OR: 1.68; 95% CI: 0.67, 4.21) compared to never users (Table 2). Time since last use did not affect the association with BC risk. ORs were 1.14 (95% CI: 0.81, 1.61) in users with time since last use of 10 years or more and 1.16 (95% CI: 0.69, 1.95) in users with time since last use under 10 years. With regard to total duration of use, multivariate adjusted ORs were 1.53 (95% CI: 0.76, 3.08) among women who used HC for ten years or more and 1.15 (95% CI: 0.84, 1.58) among women who used HC for less than ten years as compared to never users. When comparing types of HC, a borderline effect on risk was observed with injected or transdermal (Algestone/Estradiol) treatment as compared to never users (OR: 1.79; 95% CI: 0.91, 3.53). Further adjustment for admixture variables showed a slight increase of the associations (Table 2). No modification of effect was observed when data were stratified by BMI (*P* for interaction between HC and BMI  =  0.68). There was no interaction between HC and admixture (*P*  =  0.53) (data not shown).

**Table 2:** OR (95% CI) of breast cancer in pre-menopausal women according to the use of hormonal contraceptive treatment.

		Age adjusted	Multivariate Adjusted*	Multivariate Adjusted**
	Case/Control	OR, 95% CI	OR, 95% CI	OR, 95% CI
Never user	216/253	1.00 (reference)	1.00 (reference)	1.00 (reference)
Ever user	199/223	1.06 (0.82–1.38)	1.11 (0.82–1.49)	1.22 (0.91–1.65)
*Time since last use*				
Never	216/253	1.00 (reference)	1.00 (reference)	1.00 (reference)
> 10 years	120/134	0.98 (0.71–1.36)	1.14 (0.81–1.61)	1.18 (0.82–1.68)
< 10 years	37/39	1.04 (0.63–2.72)	1.16 (0.69–1.95)	1.31 (0.77–2.23)
recent (***)	12/9	1.58 (0.65–3.86)	1.68 (0.67–4.21)	1.93 (0.76–4.88)
*P* trend		0.59	0.22	0.09
*Total duration of use*				
Never	216/253	1.00 (reference)	1.00 (reference)	1.00 (reference)
< 10 years	149/164	1.00 (0.74–1.34)	1.15 (0.84–1.58)	1.20 (0.87–1.67)
> 10 years	22/19	1.39 (0.72–2.71)	1.53 (0.76–3.08)	1.79 (0.88–3.65)
*P* trend		0.36	0.12	0.05
*Type of treatment*				
Never	216/253	1.00 (reference)	1.00 (reference)	1.00 (reference)
Oral	47/61	0.90 (0.58–1.39)	0.94 (0.59–1.49)	0.92 (0.58–1.48)
Injected or transdermal	27/23	1.54 (0.83–2.85)	1.79 (0.91–3.53)	1.92 (0.96–3.84)
Others	9/13	0.86 (0.35–2.07)	0.99 (0.39–2.54)	0.99 (0.38–2.59)
More than one treatment	40/51	0.85 (0.53–1.36)	0.94 (0.57–1.57)	1.02 (0.61–1.71)

(*) Adjusted for: age, socioeconomically status (high/medium/low), BMI (kg/m2), familial history of breast cancer in first degree relatives (yes/no), diabetes(yes/no), number of full term pregnancy, age at first full term pregnancy (years), total duration of breast feeding (months) and age at menarche (years).

(**) additional adjusted for European ancestry.

(***) We defined the recent use as the reported use of contraceptive treatment during the year prior to the date of interview.

### HRT use and breast cancer risk in post-menopausal women

In multivariate models, ever use of HRT was associated with a significant increase in BC (OR  =  1.45; 95% CI: 1.01, 2.08) (Table 3). Both recent and past use showed a borderline significant association. The multivariate adjusted ORs were 1.86 (95% CI: 0.97, 3.55) for recent use and 1.45 (95% CI: 0.97, 2.17) for past use as compared to never use. Duration of use of less than 5 years was significantly associated with BC risk (OR  =  1.48; 95% CI: 1.01, 2.17). Whereas the association was stronger for 5 or more years of use, it did not reach statistical significance, possibly because of the small sample size in this category (OR  =  1.95; 95% CI: 0.90, 4.26). Types of treatment were classified as estrogen alone and combined treatment (estrogen/progestin). Although non-significant in both cases, the magnitude of the effect was higher for combined treatment (OR: 1.85; 95% CI: 0.84, 4.07) than for usage of estrogen alone (OR  =  1.14; 95% CI: 0.68, 1.91) (Table 3). When taking BMI into account, a significant association was observed among overweight women (BMI > 25 kg/m^2)^) who ever used HRT as compared to never users (OR  =  1.53; 95% CI: 1.06, 2.22), but not in normal weight women (OR  =  1.13; 95% CI: 0.36, 3.53) (Table 4). Results were not affected after stratifying for admixture variables (*P* for interaction between HRT and admixture  =  0.27) (data not shown).

**Table 3:** OR (95% CI) of breast cancer in post-menopause women according to the use of hormone replacement therapy.

		Age adjusted	Multivariate Adjusted*	Multivariate Adjusted**
	Case/Control	OR (95% CI)	OR (95% CI)	OR (95% CI)
Never user	419/478	1.00 (reference)	1.00 (reference)	1.00 (reference)
Ever user	133/87	**1.78 (1.32**–**2.41)**	**1.45 (1.01**–**2.08)**	**1.41((1.01**–**1.99)**
*Time since last use*				
Never	419/478	1.00 (reference)	1.00 (reference)	1.00 (reference)
Past	89/56	**1.60 (1.10**–**2.32)**	1.45 (0.97–2.17)	1.40 (0.93–2.13)
Recent (***)	35/17	**2.19 (1.19**–**4.03)**	1.86 (0.97–3.55)	1.82 (0.94–3.54)
*P* trend		**0.0009**	**0.02**	**0.03**
*Total duration of use*				
Never	419/478	1.00 (reference)	1.00 (reference)	1.00 (reference)
< 5 years	96/63	**1.54 (1.08**–**2.20)**	**1.48 (1.01**–**2.17)**	1.42 (0.96–2.11)
> 5 years	29/11	**2.79 (1.36**–**5.73)**	1.95 (0.90–4.26)	1.96 (0.87–4.39)
*P* trend		**0.006**	0.07	0.10
*Type of treatment*				
Never	419/478	1.00 (reference)	1.00 (reference)	1.00 (reference)
Estrogen alone	44/34	1.36 (0.84–2.20)	1.14 (0.68–1.91)	1.17 (0.69–2.00)
Estrogen/Progestin	22/12	**2.26 (1.07**–**4.80)**	1.85 (0.84–4.07)	1.89 (0.84–4.29)

(*) Adjusted for: age, socioeconomic status (high/medium/low), BMI (kg/m2), familial history of breast cancer in first degree relatives (yes/no), diabetes(yes/no), number of full term pregnancy, age at first full term pregnancy (years), total duration of breast feeding (months) and age at menarche (years).

(**) additional adjusted for European ancestry.

(***) We defined the recent use as the reported use of menopausal hormone therapy during the year prior to the date of interview.

**Table 4:** OR (95% CI) of breast cancer in premenopausal women according to ever use of hormone contraceptive, and in postmenopausal women according to ever use of hormone replacement therapy, stratified by BMI.

	Premenopausal				Postmenopausal			
	BMI < 25 *		BMI ≥ 25*		BMI < 25 *		BMI ≥ 25*	
	Case/control	OR (95% CI)	Case/control	OR (95% CI)	Case/control	OR (95% CI)	Case/control	OR (95% CI)
Never use	51/45	1.00 (reference)	165/208	1.00 (reference)	64/56	1.00 (reference)	355/422	1.00 (reference)
Ever use	49/29	1.50 (0.63–3.56)	150/194	1.03 (0.74–1.43)	21/13	1.13 (0.36–3.53)	112/74	**1.53 (1.06**–**2.22)**
Never	51/45	1.00 (reference)	165/208	1.00 (reference)	64/56	1.00 (reference)	355/422	1.00 (reference)
Recent	2/1	1.15 (0.08–16.26)	10/8	1.78 (0.67–4.77)	7/3	1.22 (0.21–7.03)	28/14	**2.03 (1.00**–**4.14)**
Past	40/23	1.60 (0.64–4.02)	117/150	1.08 (0.75–1.53)	12/5	**5.6 (1.07**–**29.51)**	77/51	1.47 (0.96–2.25)

Adjusted for: age, socioeconomic status (high/medium/low), BMI (kg/m2), familial history of breast cancer in first degree relatives (yes/no), diabetes(yes/no), number of full term pregnancy, age at first full term pregnancy (years), total duration of breast feeding (months) and age at menarche (years)

## Discussion

The purpose of this study was to bring a better understanding of the effect of HC and HRT on BC risk among Mexican women. Among premenopausal women, ever and recent users of HC had a non-significant increase in BC risk compared to never users. In postmenopausal women, an increase in BC risk was observed among HRT users. The effect is considerably more important for current use, for combined progestin/estrogen use, and for 5 or more years of use than for past use, estrogen only, and duration of use less than 5 years, respectively.

The potential association between HC and BC risk has been investigated in a number of epidemiological studies [7], [8], [10], [11], [30]–[32]. They often reported that recent use of HC, as well as long duration, was associated with an increased risk of BC. In premenopausal Hispanic women, Sweeny *et al.* [25] reported a non-significant increased risk of BC among recent users of HC compared to never users (OR  =  1.22; 95% CI: 0.80, 1.84 for oral contraceptives). In our study, the highest increased risk was observed in the class of injected contraceptive (OR was 1.79; 95% CI: 0.91, 3.53) despite the small size of the subgroup (cases/controls  =  27/23). In the largest meta-analysis including 54 epidemiologic studies, women currently using HC had a modestly elevated risk for BC. This risk continuously decreased with years of treatment cessation and became null after ten years (RRs: in current users  =  1.24 (95% CI: 1.15, 1.33); 1–4 years after stopping  =  1.16 (95% CI: 1.08, 1.23), 5–9 years after stopping  =  1.07 (95% CI: 1.02, 1.13) and 10 or more years after stopping  =  1.01 (95% CI: 0.96, 1.05)) [7]. In our study, the time since last use and the total duration of use did not affect the association with BC risk; however the size of many of the subgroups was too small to provide an accurate estimate of the effect.

Our findings reported for HRT in relation to BC risk among postmenopausal women are essentially compatible with published epidemiological studies [17], [19], [21], [33]–[37]. In a nested case-control study, current HRT users have been found to have a higher risk of BC than never-users, adjusted OR  =  2.1 (95% CI: 1.5, 3.0). In the Million Women Study [38], current use of HRT was associated with an increase in BC risk and was significantly different from the risk associated with past use. The use of combination HRT (estrogen and progestin) for more than 5 years resulted in the highest risk of BC, OR  =  3.0 (95% CI: 1.9–4.7) [21]. The WHI (Women’s Health Initiative) reported that the magnitude of the effects for combined estrogen/progestin treatment was higher than for the usage of estrogen treatment alone [19], [22]. Similarly, Lee *et al* reported that current estrogen-progestin therapy use was associated with a 29% increased risk of BC per 5 years of use (95% CI: 23, 35%), and current estrogen therapy use with a 10% increase in risk per 5 years of use (95% CI  =  5–16%) [20]. Additionally, one recent cohort study reported that estrogen plus progestin was associated with more invasive BC compared with placebo (hazard ratio (HR): 1.25; 95% CI: 1.07–1.46; *P*  =  0.004) [9]. However, few of these studies have assessed associations among Hispanic populations. Li *et al.* [39] reported that Hispanic postmenopausal women appeared to be at a significantly greater risk from HRT than non-Hispanic white women (OR for estrogen treatment for longer than 140 months in Hispanics: 5.53 (95%CI: 1.47–20.87); in non-Hispanic whites: 2.65 (95%CI: 0.95–7.34), compared with estrogen treatment for less than 17 months). In a multiethnic cohort study [20], Latino women who used estrogen/progestin treatment had a 36% increased risk of BC per 5 years of use (OR  =  1.36; 95%CI: 1.20, 1.54), whereas no significant association was observed with the use of estrogen alone.

Our study confirms and strengthens previously published results. In particular, this study is the largest population based case-control of hormonal therapy and BC in Mexico. The documentation of established BC risk factors in our study argues against serious bias. Recall bias is unlikely given the lack of awareness among women in our population of possible links between hormonal treatment and BC risk. SES status could be a potential confounding factor; however there is no evidence of significant interaction between SES and HRT, or between SES and HC. Another potential limitation of our study is the relative low prevalence of HRT users in this population (19.7%), leading to a lack power for some analyses and thus reducing the accuracy of the risk estimates. Mexico is a country in economical and epidemiological transition where chronic diseases are increasing rapidly. Given the lack of public awareness and of adequate public health measures, the use of hormonal therapy will probably continue to increase over the next years. Therefore these findings provide new information about the effects of HC and HRT on cancer risk that should help women make informed decisions about their choice, and should support appropriate initiatives by public health bodies.

HC and HRT usually contain the sex hormones estrogen and progesterone. These hormones have been reported to exert different effects on different tissues, but the exact mechanisms related to the risk of BC are still not completely clear [40]. Estrogens are known to increase the rate of cell division within the ductal epithelium of the breast, and hence increase the probability of mutation occurring or of promotion of an existing mutation. In addition, progesterone and progestin may augment this effect [40], [41]. Another hypothesis is that HRT use increases the radiological breast density (mammography density), a marker of change in composition of breast tissues, which is known to be a main risk factor for BC occurrence [42].

In conclusion, our results indicate that, among Mexican women, the use of HC had a non-significant effect on the risk of pre-menopausal BC, but suggested that injected contraceptives may slightly increase the risk. HRT had a significant effect on post-menopausal BC, in particular for combined hormones and long duration users. This study provides new information about the effects of HC and HRT on BC risk in this population, which may be of relevance for the population of Latin America as a whole. Further research on the impact of injected contraceptives is warranted.
